# Hematogenous dissemination of pathogenic and non-pathogenic *Leptospira* in a short-term murine model of infection

**DOI:** 10.3389/fcimb.2022.917962

**Published:** 2022-07-18

**Authors:** Matthew C. Surdel, Phillip N. Anderson, Beth L. Hahn, Jenifer Coburn

**Affiliations:** ^1^ Department of Medicine, Division of Infectious Diseases, Medical College of Wisconsin, Milwaukee, WI, United States; ^2^ Department of Microbiology and Immunology, Medical College of Wisconsin, Milwaukee, WI, United States

**Keywords:** *Leptospira*, infectious disease, pathogenic factors, tropism, murine model, adhesion

## Abstract

Leptospirosis is an emerging zoonosis caused by pathogenic *Leptospira* spp. Because rodents are natural hosts of *Leptospira*, rodent models of pathogenesis have been limited, but are valuable to understand infection in reservoir animals even in the absence of disease. Mouse models of infection provide advantages due to genetic tractability, so developing murine models of *Leptospira* infection is crucial for further understanding the biology of this organism. Previously our laboratory developed a short-term murine model of *Borrelia burgdorferi* hematogenous dissemination to investigate the role of adhesion proteins on bacterial survival and dissemination within a host. Here we adapt this model to *Leptospira*. C3H/HeJ mice are anesthetized, inoculated intravenously, and then bacteria are allowed to circulate for up to twenty-four hours. Mice are euthanized, perfused with saline, and tissues are harvested for culture and DNA purification. Bacterial burdens are determined by quantitative PCR. Reproducible burdens of bacteria were found in tissues upon inoculation with pathogens and non-pathogens, demonstrating the utility of this model to probe different *Leptospira* species and strains. Pathogenic *L. interrogans* has a significantly higher burden in blood, liver, kidney, and bladder at one-hour post-inoculation when compared to non-pathogenic *L. biflexa*. Colonization of the kidney is essential to the life cycle of pathogenic *Leptospira* in nature. Measurable burdens of non-pathogenic *L. biflexa* were found in numerous organs and live leptospires were recovered from blood samples for at least three hours post-inoculation, contrary to the previous belief that non-pathogenic leptospires are rapidly cleared. This short-term murine model of *Leptospira* hematogenous dissemination will allow for the interrogation of virulence factors potentially important for tissue colonization and evasion of host defenses, and represents a novel animal model for investigating determinants of *Leptospira* infection.

## Introduction

Leptospirosis is a zoonosis caused by pathogenic *Leptospira* spp. *Leptospira* infects animals and humans worldwide, resulting in approximately one million human cases a year and 60,000 deaths, however, this number is likely underestimated (Abela-Ridder et al., 2010; Costa et al., 2015; reviewed in Adler and de la Pena Moctezuma, 2010). Disease caused by *Leptospira* can range from asymptomatic colonization to life threating disease with considerable mortality (Abela-Ridder et al., 2010; Costa et al., 2015; Hochedez et al., 2015; Torgerson et al., 2015; reviewed in Adler and de la Pena Moctezuma, 2010; Picardeau, 2013; Haake and Levett, 2015). Despite the prevalence of disease, additional research is needed to understand the pathogenesis of this organism in a living host.

The life cycle of pathogenic *Leptospira* requires constant colonization of and passage through reservoir animals. The major reservoir for *Leptospira* is *Rattus norvegicus*, the brown rat, however, *Leptospira* can infect a wide range of host species, including dogs, horses, cattle, pigs, mice, and other rodents (Bolin, 1996; Rohrbach et al., 2005; reviewed in Ellis, 1994; Adler and de la Pena Moctezuma, 2010; Adler et al., 2014; Haake and Levett, 2015). Upon infection, *Leptospira* disseminate and colonize the proximal tubules of the kidneys and are ultimately shed in urine (reviewed in Ko et al., 2009; Adler et al., 2014). Upon excretion, *Leptospira* has a unique ability to survive in water and soil for many months (Trueba et al., 2004; Andre-Fontaine et al., 2015). *Leptospira* then infects additional hosts and their life cycle continues. Humans become accidental hosts through exposure to contaminated water or infected animals (reviewed in Ko et al., 2009; Adler et al., 2014; Haake and Levett, 2015).

Historically, much of our understanding of leptospirosis has come through observation of infected humans, dating back to the initial description of the disease as a form of jaundice (Weil, 1886). To dissect specific mechanisms of bacterial pathogenesis, animal models are needed. Numerous models have been developed over the years to study *Leptospira* pathogenesis. These models typically utilize larger rodents and other mammals, including hamsters, guinea-pigs, rats, dogs, and monkeys (Morton, 1942; Brunner and Meyer, 1949; Minette and Shaffer, 1968; De Brito et al., 1979; Thiermann, 1981; Pereira et al., 2005; Haake, 2006; Athanazio et al., 2008; Coutinho et al., 2014; reviewed in Gomes-Solecki et al., 2017). There are many limitations to these models, including the lack of ability to utilize these animals at a wide range of institutions, difficulty of working with larger animals, higher costs of research involving larger animals, and limited genetic tools available to dissect host determinants of disease (reviewed in Evangelista and Coburn, 2010).

While the perfect model to replicate human disease does not exist, mice are one of the most commonly used models for studying multiple infectious diseases (reviewed in Sarkar and Heise, 2019). Murine models of infection provide numerous advantages when studying bacterial pathogenesis. Importantly, mice share a lot of similarities to humans, making them an attractive model (Perlman, 2016; reviewed in Sarkar and Heise, 2019). In addition, mice are genetically tractable, with a wide range of inbred strains with specific mutations commercially available, allowing for dissection of host determinants of infection. Finally, their low cost and ease of use across a wide range of research institutions makes developing a murine model of leptospirosis crucial to furthering our understanding of *Leptospira* infection.

*Leptospira* is a particularly virulent group of organisms, with a mean 50% lethal dose (LD_50_) as low as three leptospires in hamsters (Silva et al., 2008; Wunder et al., 2016; reviewed in Evangelista and Coburn, 2010). In contrast, mice are a reservoir host for *Leptospira*, and therefore, do not typically exhibit disease progression similar to humans (reviewed in Gomes-Solecki et al., 2017). Therefore, studying *Leptospira* infection in mice provides a unique model in which we can understand the biology of the organism, for example the bacterial life cycle in a natural host. Numerous groups have developed mouse models of leptospirosis for these reasons. Importantly, it has been shown that the mouse strain C3H/HeJ are susceptible to disease due to a mutation in toll-like receptor 4 (TLR4), and therefore defective in their ability to recognize LPS (Pereira et al., 1998; Poltorak et al., 1998; Qureshi et al., 1999; Nally et al., 2005; Richer et al., 2015). Utilizing this mouse strain, many mouse models have been developed, ranging from colonization to lethal disease (Nally et al., 2005; Nair and Gomes-Solecki, 2020). Natural routes of infection, such as transdermal abrasion or mucosal infection, are limited due to the inability to quantify the actual number of bacteria that enter the host. Therefore, additional mouse models of infection are needed to understand and quantitatively compare pathogenic factors within *Leptospira*.

Previously our laboratory developed a short-term model of *Borrelia burgdorferi* hematogenous dissemination in mice to investigate both adhesion of bacteria to the different vascular beds in different host tissues and bloodstream survival (Caine and Coburn, 2015). In the current study we adapt this model to *Leptospira* spp. At one-hour post-inoculation, mice were found to have significantly higher bacterial burdens of pathogenic *L. interrogans* sv. Manilae compared to non-pathogenic organisms in the blood, liver, kidney, and bladder. In addition, a time course post-inoculation detected non-pathogenic *L. biflexa* sv. Patoc in multiple organs by qPCR, and live leptospires were cultured from the blood, suggesting it is able to survive at least three hours in the host, contrary to the previous belief that non-pathogens are rapidly cleared (Bonhomme et al., 2020; reviewed in Fraga et al., 2011). Taken together, we have developed a novel murine model of *Leptospira* infection to study factors important in tissue colonization and evasion of host defenses that allows the use of both loss-of-function and gain-of-function bacterial strains, and can provide new insight into the rapid dissemination of *Leptospira*.

## Materials and methods

### Ethics statement for animal use

Animals were housed according to institutional guidelines and fed and watered *ad libitum*. The Medical College of Wisconsin Institutional Animal Care and Use Committee approved all work with animals.

### Biosafety

All liquids that contacted *Leptospira* were sterilized using 10% bleach for 30 minutes. All solid materials that come into potential contact with *Leptospira* were treated with Clidox (Pharmacal Research Laboratories, 96118F) according to manufacturer’s instructions. All work with *Leptospira* was performed in a biosafety cabinet, and sealed containers were used when transporting *Leptospira* outside of a biosafety cabinet. Biosafety cabinets were sterilized by ethanol and UV irradiation following each use.

### Preparation of bacteria for inoculation into mice

*Leptospira* were counted using a Petroff-Hausser counting chamber. Cultures were centrifuged at 3,651 x g for 20 minutes at ambient temperature. Supernatants were decanted into 10% bleach. Pellets were washed once with PBS supplemented with 0.2% heat inactivated normal C3H mouse (Charles River Laboratories, Frederick, MD) serum. Organisms were resuspended in PBS + 0.2% normal mouse serum to a final density of 10^9^ spirochetes/ml. For experiments utilizing heat-inactivated leptospires, bacteria were washed once with PBS and resuspended to a final density 10^9^ spirochetes/ml. Approximately 150 µl were distributed to 1.5 ml tubes and inactivated for 30 minutes at 95°C in a heat block.

As a secondary confirmation of bacterial number, samples of inoculum were frozen and processed as described below to assess *Leptospira* concentration by genome copies using quantitative polymerase chain reaction (qPCR).

### Intravenous inoculation of mice

Seven- to nine-week old female C3H/HeJ mice (Jackson Laboratory, Bar Harbor, ME) were anesthetized *via* intraperitoneal (IP) injection of 100 mg/kg ketamine and 10 mg/kg xylazine. Anesthesia was confirmed by pedal reflex. Anesthetized mice were placed under a heat lamp to warm the tail vein. A single animal at a time was placed in a restraining device inside the biosafety cabinet to expose the tail; the tail was cleaned with 70% ethanol. Using a 27-gauge needle, 100 μl of bacterial suspension was inoculated delivering 1 x 10^8^ total bacteria into the tail vein.

For the one-hour perfusion experiments, following inoculation the mice were placed on a warming pad inside the biosafety cabinet. An additional IP dose of ketamine/xylazine cocktail was administered 40 minutes following bacterial inoculation (20 minutes prior to perfusion and organ harvest) to maintain deep anesthesia for the duration of the experiment. For the three-, six-, and twenty-four-hour time points, mice were given an additional IP dose of ketamine/xylazine cocktail 20 minutes before perfusion and harvest to induce deep anesthesia.

### Cardiac perfusion and tissue harvest

At each time point post-inoculation, cardiac puncture was performed to collect blood from anesthetized mice using a 25-gauge needle with 60 mM sodium citrate plus 40 mM citric acid as anticoagulant. Blood samples were stored on ice and later spun at 16,110 x g for 15 mins at 4°C to separate whole blood from serum; serum was discarded. A thoracotomy was performed. A small cut, acting as a drain, was made in the right atrium as previously described (Caine and Coburn, 2015). A 21-gauge x 3/4-winged infusion kit was inserted into the left side of the heart. Mice were perfused with 0.9% sterile sodium chloride at a flow rate of 1 ml/min for 6 minutes. The outflow was collected onto an absorbent pad and gauze. After 6 mins, the perfusion was stopped. Samples of lung, heart, spleen, liver, kidney, and bladder were collected. All samples were transferred to labeled 1.5 ml tubes and placed on dry ice. Tissue was stored at -80°C. All samples, including inoculum and blood samples, were processed to obtain DNA using the DNeasy Blood and Tissue Kit (Qiagen, 69504) according to manufacturer’s instructions.

### Cultures from *L. biflexa* time course

Samples of mouse blood were added to HAN medium to recover living organisms (Hornsby et al., 2020). Cultures were incubated at 37°C under 3% CO_2_ and checked weekly for four weeks to assess presence of live *Leptospira via* darkfield microscopy.

### Quantification of spirochetes by qPCR

*Leptospira* genome copies and mouse genome copies were determined *via* qPCR amplification in triplicate performed in reactions containing 100 ng template DNA, 12.5 μl of QuantiFast SYBR Green PCR Kit (Qiagen, 204057), 1 μl of 5 μM each forward and reverse primers (final concentration of 0.2 μM), and water to bring reaction to a final volume of 25 μl. Primer sets used for *Leptospira* 16s rRNA were previously described: Forward: ‘5-TAGTGAACGGGATTAGATAC-3’, Reverse: ‘5-GGTCTACTTAATCCGTTAGG-3’ (Bedir et al., 2010). Primer sets used for *Mus musculus* β-actin were previously described: Forward: ‘5-TCACCCACACTGTGCCCATCTACGA-3’, Reverse: 5’- GGATGCCACAGGATTCCATACCCA-3’ (Caine and Coburn, 2015). qPCR was performed on a Bio-Rad CFX system using the programs: 95.0°C for 5.5 min, (95.0°C for 10 s, 60.0°C [*Leptospira* spp., 16s rRNA] or 60.9°C [*Mus musculus*, β-actin] for 30 s, repeated 39 times), 95°C for 1 min, and 50°C for 1 min followed by a melt curve of 70°C to 90°C in 0.5°C increments for 10 seconds each.

Mouse standard curves were created using genomic DNA purified from C3H mouse livers using the DNeasy Blood and Tissue Kit (Qiagen, 69504). Leptospiral standard curves were created using genomic DNA isolated from *L. biflexa* sv. Patoc and *L. interrogans* sv. Manilae with the Wizard^®^ Genomic DNA Purification Kit (Promega, Cat. No. A1120). Mouse DNA was added to each *Leptospira* standard such that 100 ng of mouse DNA was present in each qPCR reaction to replicate conditions found within samples purified above. The mass of DNA used for each standard was converted to genome number using published genome size for each *Leptospira* strain (reviewed in Ko et al., 2009), or based upon the vendor’s information for *Mus musculus* (Jackson Laboratory, Bar Harbor, ME). All genomes were quantifiable using this method down to single-digit copy numbers per reaction.

*Leptospira* 16S rRNA and *Mus musculus* β-actin gene copies were determined from the standard curves using the Bio-Rad CFX system and software. β-actin gene copies were used to confirm presence of DNA in each reaction. If mouse DNA was quantified and was more than 10-fold less than expected based upon the amount of total DNA added to each qPCR reaction, data were excluded from analysis. qPCR was repeated for samples that did not amplify DNA to confirm the absence of DNA and rule out a problem with the reaction. In addition, blood samples were diluted 1:10 prior to qPCR due to the presence of an unknown inhibitor of the qPCR reaction in these samples. *Leptospira* genome number was normalized by total DNA added to each PCR reaction. For one-hour experiments, genomes were further normalized to the inoculum concentration determined by qPCR in order to compare experiments with multiple strains performed on different days. The Mann-Whitney U Test (GraphPad Prism) was used to analyze differences when two groups were compared. The Kruskal-Wallis test, comparing the mean of each column with the mean of every other column, correcting for multiple comparisons by statistical hypothesis testing, was used when comparing three groups of data.

## Results

### Intravenous inoculation leads to reproducible bacterial burdens in numerous organs

To dissect mechanisms of potential importance to infection and pathogenesis, we adapted a murine model of *B. burgdorferi* hematogenous dissemination to be used with *Leptospira* spp. (Caine and Coburn, 2015). Mice were anesthetized and inoculated by the intravenous route through the tail vein. Bacteria were allowed to circulate for one hour, after which samples were collected. Five mice were inoculated with each bacterial strain in three independent experiments to determine reproducibility of this model. Bacterial burdens in all organs were found to be similar between experiments (**Supplementary Figure 1**). However, the two independent inoculations in which the hearts were harvested had slight, yet significant difference in burdens of *L. biflexa* sv. Patoc (**Supplementary Figure 1**). Importantly, transient presence of *Leptospira* in low quantities has been shown previously in testes following infection before ultimate colonization of the kidney (Shetty et al., 2022). Therefore, the number of organisms is likely not biologically important due to the low overall burden in the heart.

Quantifying bacterial genomes in DNA harvested from blood by qPCR suggested the presence of unknown qPCR inhibitors causing atypical amplification curves in the initial reactions. Therefore, to circumvent this issue, each blood sample was diluted 10-fold prior to qPCR quantification to dilute out potential inhibitors. Although the quality of the data was increased and the bacterial burdens grouped well in each experiment, the blood samples were significantly different when comparing independent experiments for two of the pathogenic *Leptospira* infections (**Supplementary Figure 1**). Importantly, neither of these two infections was significantly different from the third, which had an intermediate number of genomes quantified. Taken together, these data suggest that the vascular beds in a variety of tissues and organs are reproducibly colonized by *Leptospira* during this short-term infection. Furthermore, except for a few outliers, the data within each individual experiment grouped well together.

### Pathogenic *Leptospira* have increased burdens in the blood, liver, kidney, and bladder

**Figure 1:**
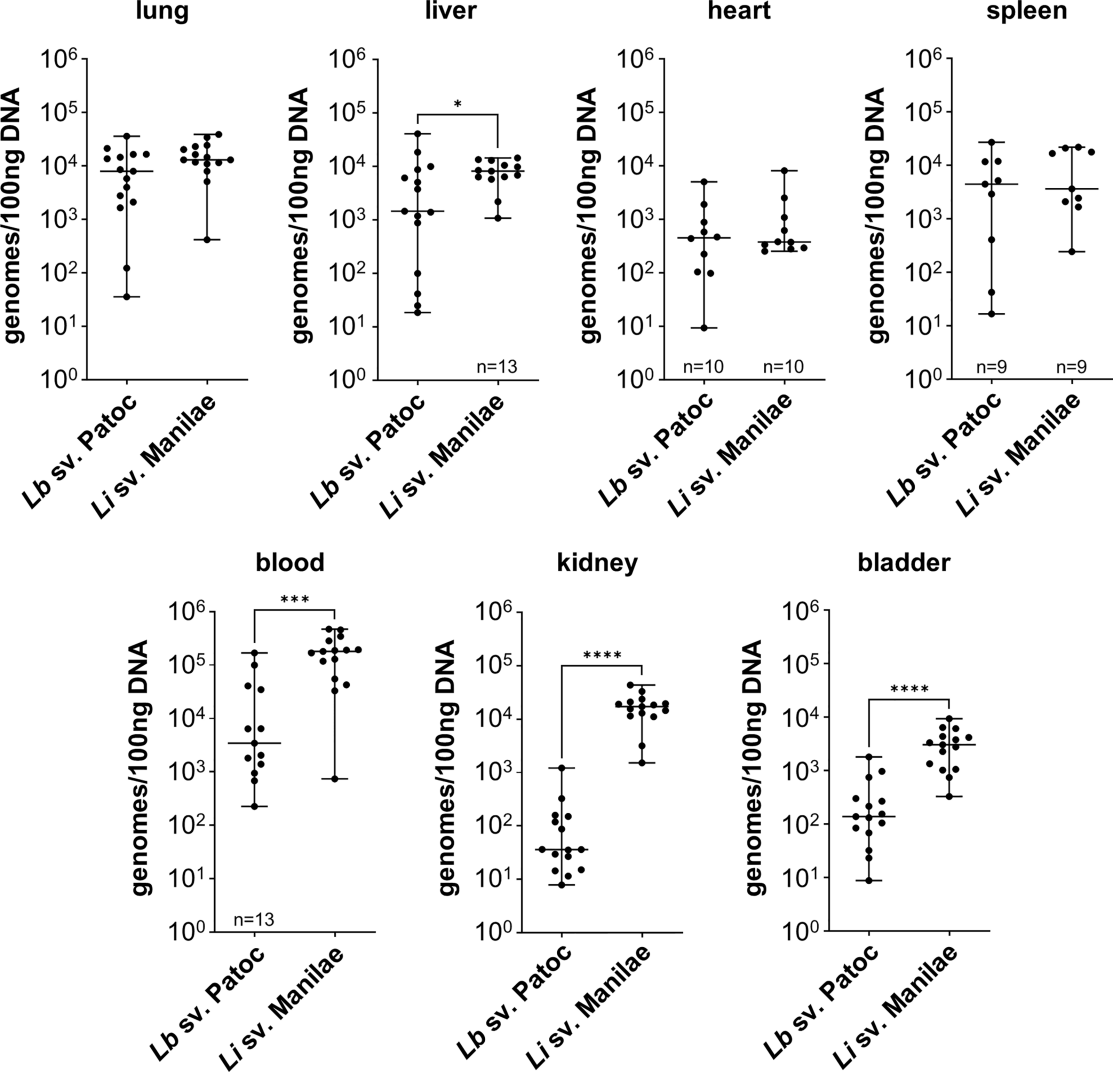
Pathogenic *Leptospira* has a tropism for the liver, kidney, and bladder. Mice were inoculated intravenously in a one-hour model of infection. DNA from tissues was harvested and quantified by qPCR. Significantly higher burdens of *L. interrogans* sv. Manilae were found in the blood, liver, kidney, and bladder compared to non-pathogenic *L. biflexa* sv. Patoc. Data represent a total of fifteen mice per group unless otherwise noted, performed as three independent experiments per strain. Heart and spleen samples were only harvested from two of the three experiments. Median ± range is plotted. * denotes *p ≤* 0.05, *** denotes *p ≤* 0.001, **** denotes *p ≤* 0.0001.

In order to determine the differences in tissue tropism between pathogenic and non-pathogenic *Leptospira*, the independent replicate experiments for each strain were combined, and strains were compared. Burdens of pathogenic *L. interrogans* sv. Manilae were significantly higher in the blood, liver, kidney, and bladder when compared to burdens of non-pathogenic *L. biflexa* sv. Patoc (**Figure 1**). These data suggests that pathogenic *Leptospira* are more resistant to clearance from the blood than are non-pathogens. As this infection is only allowed to proceed for one-hour, pathogenic *Leptospira* quickly adhere to the endothelium in the kidney and bladder, likely an initial step in colonization. Importantly, no significant differences were seen in the other organs (lung, heart, and spleen), providing evidence that the increased burdens seen in the liver, kidney, and bladder are due to specific tropism to these organs and not a general increase in bacterial load throughout the host when mice are infected with pathogenic *Leptospira*. In addition, quantifiable burdens of non-pathogenic *L. biflexa* sv. Patoc were found in all tissues tested, suggesting that non-pathogens can survive in the host and colonize tissues for at least one hour post infection (**Figure 1**).

Non-pathogenic *Leptospira* spp. are thought to be rapidly cleared from the host, however, the detection of *L. biflexa* sv. Patoc by qPCR in organs at one hour post infection begged the question of whether the DNA quantified are from living or dead organisms. Therefore, mice were inoculated with heat-inactivated *L. biflexa* sv. Patoc or heat-inactivated *L. interrogans* sv. Manilae. In the majority of tissues, quantification of bacterial burdens suggested an active process as heat-inactivated *Leptospira* were found in reduced numbers in these organs (**Supplementary Figure 2**). The kidney and bladder did not show greater burdens when infected with living *L. biflexa* sv. Patoc compared to heat-inactivated leptospires. Taken together, these data suggest that at *L. interrogans* sv. Manilae has increased ability to survive in the blood and increased tropism for the liver, kidney, and bladder following hematogenous dissemination, and that *L. biflexa* sv. Patoc is able to survive for at least one hour post infection within the host and actively adhere to multiple tissues.

### Non-pathogenic leptospires survive in the mouse for at least three hours post infection

**Figure 2:**
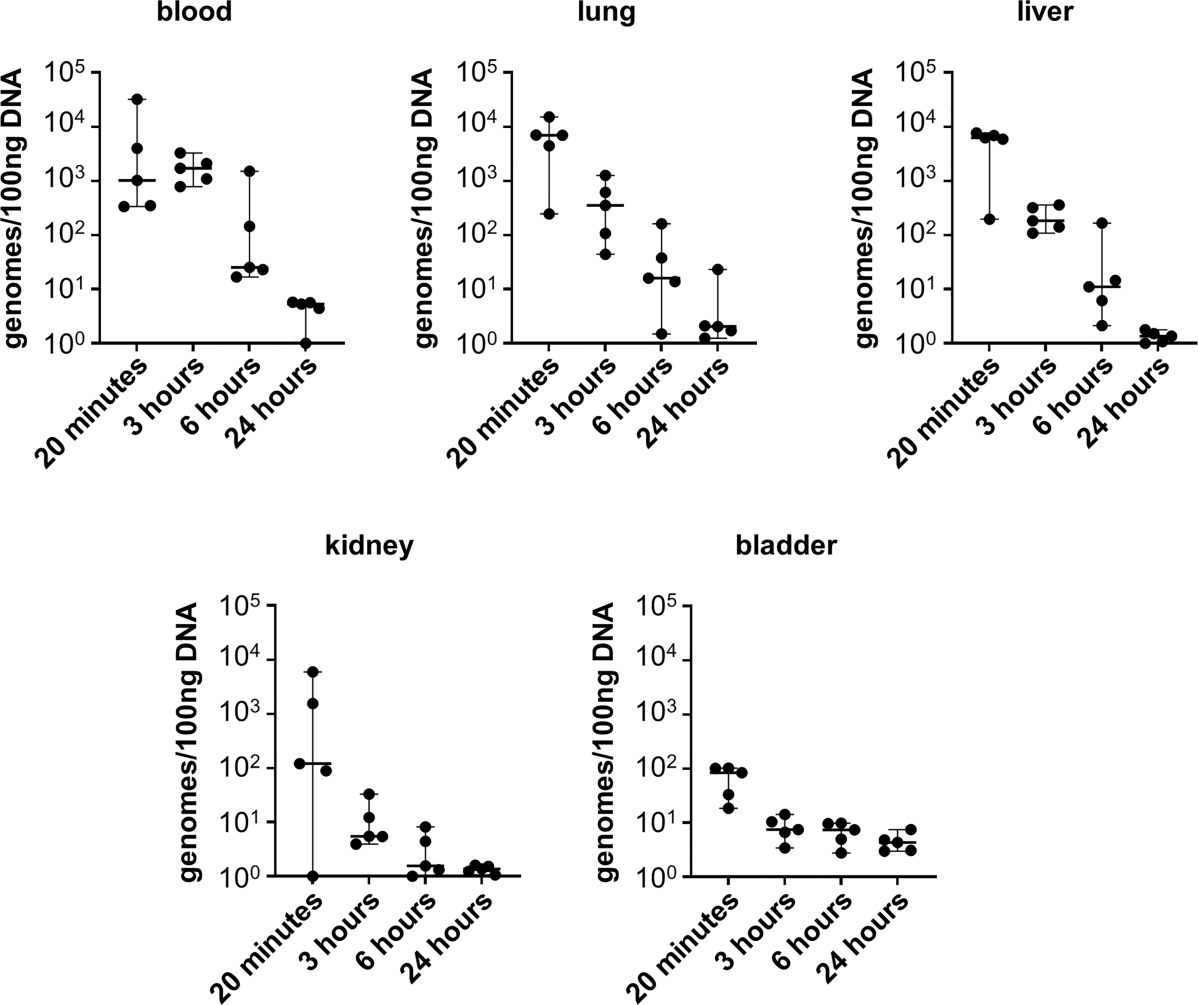
Non-pathogenic *Leptospira* is detectible for at least six hours post infection. Mice were inoculated intravenously and euthanized at various time points post-infection. DNA from tissues was harvested and quantified by qPCR. DNA was detected in significant quantities up to six hours post infection, and in some organs for twenty-four hours post infection. Data represent a total of five mice per group. Median ± range is plotted.

Given that infection with *L. biflexa* sv. Patoc led to detectable quantities of DNA, we aimed to define how long the non-pathogen could survive in the host. Mice were infected with *L. biflexa* sv. Patoc and infection was allowed to proceed for up to 24 hours. Organs were harvested and bacterial burdens quantified by qPCR. In addition, blood was cultured to determine the viability of leptospires in the host. Detectible levels of *L. biflexa* sv. Patoc were identified up to 24 hours by qPCR in most organs, however, the biological relevance of these burdens remains to be elucidated, as they may represent bacterial debris from dead or dying leptospires late in infection (**Figure 2**). Importantly, a vast majority of bacterial DNA is cleared by six hours post-infection, correlating with reduced recovery of living leptospires from blood in culture. Living bacteria were recovered from all mice at three hours post-inoculation, whereas only one mouse out of five was found to be positive at six hours post-inoculation (**Table 1**). Taken together, these data define a window of between three and six hours in which non-pathogenic *L. biflexa* sv. Patoc can survive in the host in this hematogenous dissemination murine model of *Leptospira* infection.

**Table 1:** Culture positive blood samples from time course with *L. biflexa* sv. Patoc.

culture positive/total mice (% positive)			
20 minute	3 hour	6 hour	24 hour
5/5 (100)	5/5 (100)	1/5 (20)	0/5 (0)

## Discussion

Dissecting mechanisms of bacterial pathogenesis requires the use of animal models. Models previously developed for *Leptospira* infection typically utilize larger rodents, such as hamsters (Morton, 1942; Brunner and Meyer, 1949; Minette and Shaffer, 1968; De Brito et al., 1979; Thiermann, 1981; Pereira et al., 2005; Haake, 2006; Athanazio et al., 2008; Coutinho et al., 2014; reviewed in Gomes-Solecki et al., 2017). Although they exhibit acute disease similar to that of humans, fewer tools are available for these animals to dissect specific mechanisms. Mice, on the other hand, would provide novel insight into the pathogenesis of *Leptospira* due to their genetic tractability and numerous inbred strains with varying genotypes that are widely available. In addition, mice represent a reservoir host for *Leptospira* infection, and studying colonization in a mouse model will provide important insight into the natural life cycle of *Leptospira*. Therefore, development of mouse models of *Leptospira* infection is important to understanding leptospirosis.

Previously, our laboratory developed a short-term model of *B. burgdorferi* hematogenous dissemination to interrogate adhesion to and colonization of host tissues, as well as evasion of host defenses (Caine and Coburn, 2015). In the current study, we adapted this model to study *Leptospira* spp. infection. Mouse strains exhibit differential susceptibility to infection by *Leptospira*. C3H/HeJ mice, a strain known to be susceptible to *Leptospira*, were selected for use in our short-term model of infection (Poltorak et al., 1998; Nally et al., 2005; da Silva et al., 2012; Richer et al., 2015; Shetty et al., 2021; Shetty et al., 2022). It has long been known that TLR4 is important in controlling leptospirosis, as mice lacking TLR4 are susceptible to acute disease (Pereira et al., 1998; Viriyakosol et al., 2006; Nair et al., 2020a). C3H/HeJ mice have a spontaneous proline to histidine mutation at residue 712 which produces a hyporesponsive TLR4, leading to susceptibility to *Leptospira* infection as well as limiting endotoxic shock induced by lipopolysaccharide (LPS) (Qureshi et al., 1999). This limitation of the endotoxic shock response, especially when a large number of bacteria are introduced directly into the bloodstream, is more humane. In addition, *Leptospira* spp. encode a unique LPS that leads to varied recognition by TLRs in humans and mice (Que-Gewirth et al., 2004; Nahori et al., 2005; Chassin et al., 2009). Importantly, the presence of either mouse or human TLR4 has been shown to be sufficient to control infection by *Leptospira* (Nair et al., 2020a). In addition to mouse strains, age, sex, and the route of infection contribute to variable disease kinetics induced by *Leptospira* (Pereira et al., 1998; Nally et al., 2005; Nair et al., 2020b; reviewed in Gomes-Solecki et al., 2017). Importantly, C3H/HeJ mice nine-weeks of age have been shown to be susceptible to disease when infected intraperitoneally with *L. interrogans* (Koizumi and Watanabe, 2003). Whereas natural routes of infection lead to variable levels of bacterial entry into the host, intravenous inoculation allows for standardization of the inoculum and therefore quantification and statistical analyses of resulting phenotypes. For these multiple reasons, we pursued a hematogenous dissemination model of *Leptospira* infection in the C3H/HeJ mouse strain. Mice were inoculated intravenously with either non-pathogenic *L. biflexa* sv. Patoc or pathogenic *L. interrogans* sv. Manilae in three independent experiments per strain. Comparable burdens were quantified in most organs between the three infections (**Supplementary Figure 1**). In addition, within each experiment bacterial burdens in organs generally grouped together, further confirming the utility of this model in studying *Leptospira* pathogenesis. These data suggest that the short-term model of *Leptospira* hematogenous dissemination warranted further investigation.

During the natural life cycle of *Leptospira*, bacteria infect and disseminate to the proximal tubules of the kidneys, likely through the blood (reviewed in Ko et al., 2009; Adler et al., 2014). Infection of mice with pathogenic *L. interrogans* sv. Manilae led to significantly higher burdens in the blood, liver, kidney, and bladder when compared to non-pathogenic *L. biflexa* sv. Patoc (**Figure 1**). Infection was only allowed to proceed for one hour, suggesting pathogens rapidly adhere to the endothelium of the kidney, a likely step in colonization. Although the higher burdens in the kidney are consistent with the known life cycle of pathogenic *Leptospira*, our model could not differentiate between colonization of the bladder following filtration in the kidney, or a specific tropism to the bladder after intravenous inoculation. Future studies will be needed to dissect the order of events leading to increased burdens of the pathogen compared to the non-pathogen in the bladder.

It is widely believed that non-pathogenic *Leptospira* are rapidly cleared within a living host (Bonhomme et al., 2020; reviewed in Fraga et al., 2011). In the one-hour model of infection, non-pathogenic *L. biflexa* sv. Patoc was detected by qPCR in all tissues, suggesting survival and hematogenous dissemination of the non-pathogen (**Figure 1**). Importantly, heat-inactivated *L. biflexa* sv. Patoc did not exhibit the same adherence to tissues, suggesting an active process by living leptospires in the host (**Supplementary Figure 2**). Therefore, it was important to determine the specific amount of time non-pathogenic *Leptospira* is able to survive in the murine host. A time course was performed in which mice were inoculated with *L. biflexa* sv. Patoc and harvested at one, three, six, or twenty-four hours post-inoculation. Importantly, *L. biflexa* sv. Patoc DNA was quantifiable at most time points post infection, however, a large decrease in burdens were seen by six hours (**Figure 2**). This decline in bacterial burden correlates with a decrease in culture positive mice between three and six hours post infection (**Table 1**). Taken together, we have defined the ability of *L. biflexa* sv. Patoc to survive in mice for between three and six hours post infection.

These results help to explain recent studies published regarding the immune response to *L. biflexa* sv. Patoc. Specifically, a significant immune response is induced by non-pathogenic *Leptospira* and can be detected at 24 hours post infection, however, this did not correlate with the presence of living leptospires in the animal (Shetty et al., 2021; Kundu et al., 2022). Consistent with this, we have shown that *L. biflexa* survives and disseminates for between three and six hours when inoculated intravenously. Although this is not the natural route of infection, it is known that *Leptospira* disseminates through the blood during infection. Thus, the immune response that has previously been detected at twenty-four hours post infection may be due to the transient presence of *L. biflexa* sv. Patoc in this newly defined time frame.

Until recently, genetic tools to dissect pathogenic virulence factors have been limited (Aviat et al., 2010; Pappas et al., 2015; Fernandes et al., 2021; reviewed in Ko et al., 2009). Despite the advent of novel genetic tools, pathogenic strains of *Leptospira* remain difficult to genetically modify due to low levels of homologous recombination. Libraries of transposon mutants in pathogenic strains have been created, but remain incomplete due to lack of saturation of the genome (Bourhy et al., 2005; Murray et al., 2009; Slamti and Picardeau, 2012). The genetic tools developed, such as transformable plasmids driving expression of specific genes, are more easily utilized in non-pathogenic strains (Pappas et al., 2015; Gaultney et al., 2020). Because of this, non-pathogens are useful tools to understand the role of specific pathogenic virulence factors by heterologous expression through gain-of-function (GOF) mutations. The short-term model of hematogenous dissemination described here allowed for quantification of non-pathogenic *Leptospira* in numerous organs, suggesting future GOF mutants in non-pathogens could be interrogated for their role in tissue tropism, as was the case for *B. burgdorferi* (Caine and Coburn, 2015). This is important because previous models have focused on later time points, when *L. biflexa* has been cleared. Thus, we present a novel method by which specific pathogenic factors can be interrogated in the context of a non-pathogen to determine their role in pathogenesis of *Leptospira*.

This short-term model of hematogenous dissemination has shown utility in adding to our current understanding of the pathogenic factors important for *Leptospira* infection. Specifically, this model allows for reproducible bacterial burdens to be detected in multiple tissues and using this model we have shown that pathogenic strains quickly amass in organs known to be involved in the natural life cycle of *Leptospira*. Therefore, this model can be applied in future studies to dissect specific pathogenic mechanisms of *Leptospira* in dissemination and evasion of host defenses. Either *L. interrogans* mutants lacking expression of potential virulence factors, or *L. biflexa* GOF mutants can be tested to determine the specific effects of genes involved in pathogenesis, as described in a separate manuscript published in this issue of *Frontiers in Cellular and Infection Microbiology* (Surdel et al., 2022). In addition, this model could be adapted to the many widely available mouse strains with specific genetic mutations, providing the exciting possibility of a new method for identifying not only bacterial factors required for pathogenesis, but also host factors that contribute to pathogenesis. In conclusion, we have developed a novel model of *Leptospira* infection that can be applied to further understand the pathogenic factors of *Leptospira* in a reservoir host, and defined the length of time a non-pathogen can survive within the murine host.

## Data availability statement

The original contributions presented in the study are included in the article/**Supplementary Material**. Further inquiries can be directed to the corresponding author.

## Ethics statement

The animal study was reviewed and approved by Institutional Animal Care and Use Committee, Medical College of Wisconsin.

## Author contributions

MS, PA, BH, and JC designed the experiments. PA and BH performed experiments. MS and PA performed analysis. MS and JC interpreted the data. MS wrote the first draft of this paper. PA and BH wrote sections of the manuscript. JC edited the manuscript. All authors contributed to the article and approved the manuscript for submission.

## Funding

This work was funded by grants R21AI147573, R01AI118799, and R01AI121217 from the National Institutes of Health, National Institute of Allergy and Infectious Diseases.

## Conflict of interest

The authors declare that the research was conducted in the absence of any commercial or financial relationships that could be construed as potential conflicts of interest.

## Publisher’s note

All claims expressed in this article are solely those of the authors and do not necessarily represent those of their affiliated organizations, or those of the publisher, the editors and the reviewers. Any product that may be evaluated in this article, or claim that may be made by its manufacturer, is not guaranteed or endorsed by the publisher.
