# Supplementary material for: Hematogenous dissemination of pathogenic and non-pathogenic Leptospira in a short-term murine model of infection
Source: Front Cell Infect Microbiol. 2022 Jul 18;12:917962. doi: 10.3389/fcimb.2022.917962 (PMC9339599; doi:10.3389/fcimb.2022.917962)
Supplement: Supplementary file 1 [file DataSheet_1.pdf]

Supplementary Material

1.1 Supplementary Figures

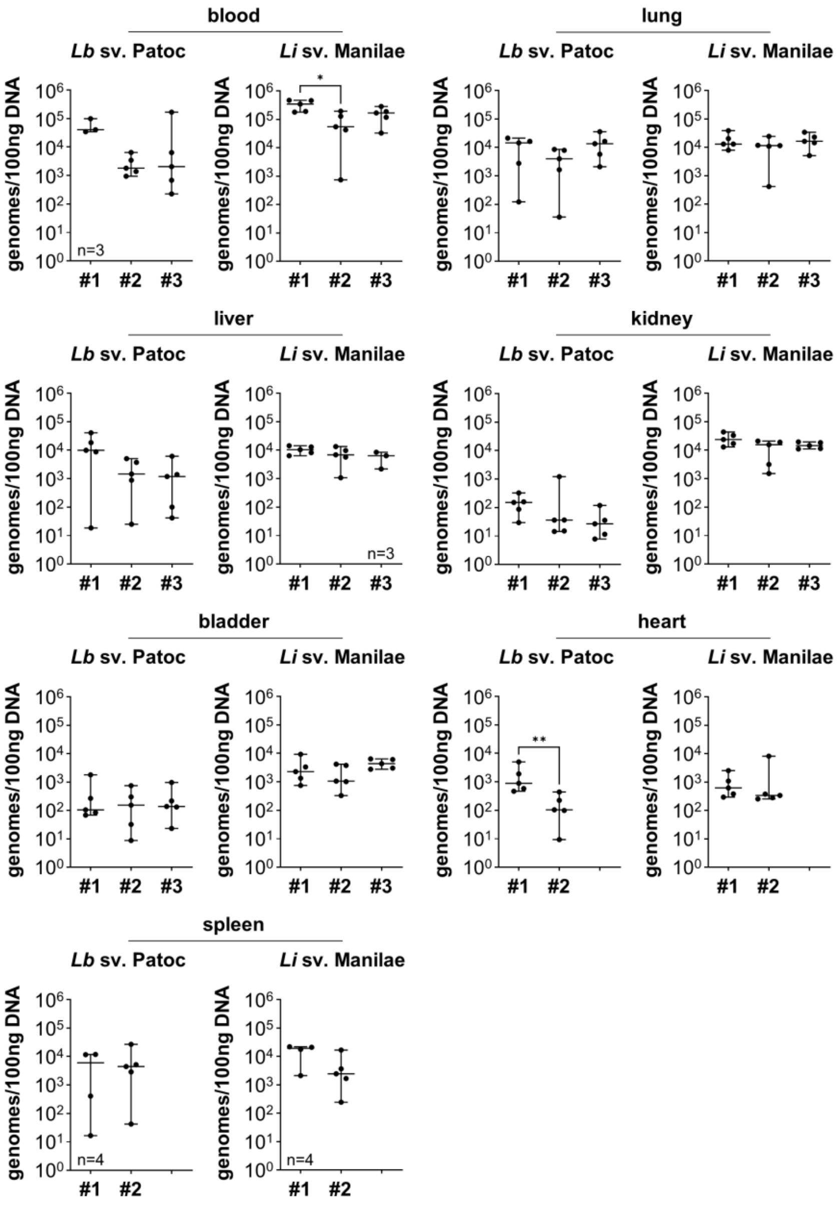

Supplementary Figure 1. Bacterial burdens quantified from independent experiments. Mice were inoculated intravenously in a one-hour model of infection. DNA from organs was harvested and quantified by qPCR. Slight differences were seen in the heart and blood, however, in general data show reproducibility between independent experiments. Data represent a total of five mice per group unless otherwise noted, performed in three independent experiments. Heart and spleen were only harvested during two of the three experiments. Median ± range is plotted. * denotes $p \leq 0.05$, ** denotes $p \leq 0.01$.

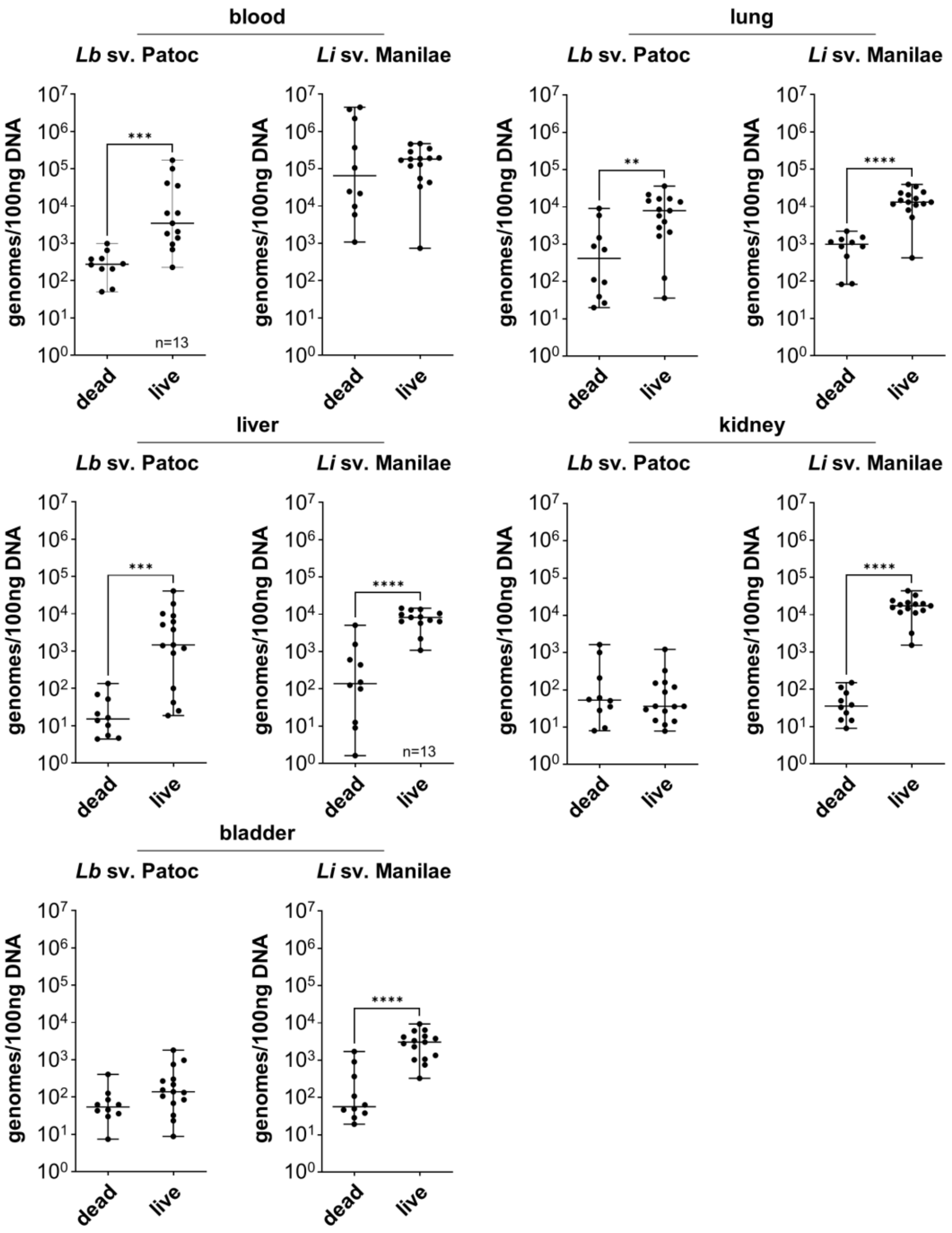

Supplementary Figure 2. Live *Leptospira* disseminates and colonizes host tissues. Mice were inoculated intravenously in a one-hour model of infection with living or heat-inactivated leptospires. DNA from tissues was harvested and quantified by qPCR. Living non-pathogenic leptospires had significantly higher burdens compared to heat-inactivated leptospires in the blood, lung, and liver. Living pathogenic leptospires had significantly higher burdens compared to heat-inactivated leptospires in the lung, liver, kidney, and bladder. Data represent a total of fifteen mice per group performed as three independent experiments per strain for living bacteria, or ten mice per group performed as two independent experiments per strain for heat-inactivated bacteria. Median ± range is plotted. ** denotes $p \leq 0.01$, *** denotes $p \leq 0.001$, **** denotes $p \leq 0.0001$.
